# Sexual distress and quality of life among women with bipolar disorder

**DOI:** 10.1186/s40345-017-0098-0

**Published:** 2017-06-06

**Authors:** Thea Sørensen, A. Giraldi, M. Vinberg

**Affiliations:** 1 Copenhagen Affective Disorder Clinic, Psychiatric Centre Copenhagen, Copenhagen University Hospital, Rigshospitalet, Dep. 6233, Blegdamsvej 9, 2100 Copenhagen, Denmark; 2 Sexological Clinic, Psychiatric Centre Copenhagen, Copenhagen University Hospital, Copenhagen, Denmark; 3 Department of Clinical Medicine, University of Copenhagen, Copenhagen, Denmark

**Keywords:** Sexual distress, Sexual functioning, Quality of life, Bipolar disorder

## Abstract

**Background:**

Information on the association between bipolar disorder (BD), sexual satisfaction, sexual function, sexual distress and quality of life (QoL) is sparse. This study aims, in women with BD, to (i) investigate sexual dysfunction, sexual distress, general sexual satisfaction and QoL; (ii) explore whether sexual distress was related to affective symptoms and (iii) investigate whether QoL was associated with sexual distress. The study is a questionnaire survey in an outpatient cohort of women with BD using: Changes in Sexual Functioning Questionnaire, Female Sexual Distress Scale, Altman Self-Rating Mania Scale (ASRM), Major Depression Inventory (MDI) and The World Health Organisation Quality of Life-Brief.

**Results:**

In total, 61 women (age range 19–63, mean 33.7 years) were recruited. Overall, 54% reported sexual distress (*n* = 33) and 39% were not satisfied with their sexual life (*n* = 24). Women with BD were significantly more sexually distressed in comparison with Danish women from the background population but they did not have a higher prevalence of impaired sexual function. Better sexual function was positively associated with ASRM scores while MDI scores were associated with more distress. Finally, the group of non-sexually distressed women with BD reported higher QoL scores compared with the sexually distressed group.

**Conclusions:**

Women with BD exhibited a high prevalence of sexual distress and their sexual function seemed associated with their actual mood symptoms and perception of QoL.

## Background

Sexual dysfunction is defined as an impaired sexual function that causes distress. The definition has changed several times over the course of time but a division into four categories overall has remained: desire/interest, arousal, orgasm and pain disorders (Basson et al. 2000, 2004, 2010; McCabe et al. 2016). Sexual dysfunction is caused by biological, psychological and social interactions and factors with negative influence on human well-being. Additionally, the risk of sexual dysfunction is increased by factors such as socio-economic status (Christensen et al. 2011a), psychiatric disorders (Brotto et al. 2011), partner status and the length of relationship (Hayes et al. 2008; Eplov et al. 2007), menopause (Ornat et al. 2013) and the side effects of psychotropic treatment (Bergh and Giraldi 2014; Serretti and Chiesa 2011a; Zemishlany and Weizman 2008). A Danish study (*n* = 4415) concluded that mental health problems and poor self-rated health problems were strongly associated with female sexual dysfunction (Christensen et al. 2011b).

Information on the association between bipolar disorder, quality of life, sexual satisfaction, sexual function and distress is sparse. It is well known that depression and antidepressants affect sexual function negatively (Clayton et al. 2014), but only a few studies include women with BD. Besides, the studies have small study populations and different questionnaires, none of which include sexual distress (Dell’Osso et al. 2009; Ghadirian et al. 1992; Grover et al. 2014). In an Italian study (Dell’Osso et al. 2009) comparing 142 patients (60 with BD and 82 with unipolar depression) with 101 healthy controls, patients with BD reported more sexual dysfunction compared to healthy control persons. Patients with BD had increased sexual desire in comparison with patients with unipolar disorder in the Italian study. Also, the presence of periods characterised by frequent sexual partners was significantly associated with the feeling that life is not worth living and sexual dysfunction was associated with lifetime suicide attempts. Finally, a Dutch study in the general population of the Netherlands showed an association between BD and sexual dissatisfaction (Vanwesenbeeck et al. 2014).

Episodes of depression or mania can be trigged by stress. Patients with BD are probably more easily distressed, and therefore, sexual distress could be a potential trigger for depression and/or mania. The possible relationship between sexual distress and affective symptoms in patients with BD has not been evaluated previously. Further, quality of life (QoL), the general well-being and observed life satisfaction in many aspects—for example, physical and psychological health, education, employment, wealth, finance, environment, social relations and sexual function—are important aspects of QoL. Patients with affective disorders have a lower score of QoL compared to the general population (Nørholm 2008; Yatham et al. 2004; Nørholm and Bech 2001). When measuring QoL, according to World Health Organisation, sexual satisfaction is included as part of the total score (WHOQOL Group 1998). To improve treatment and QoL clinically in patients with BD, it is thus important to also focus on and include sexual function. The aim of the present study was, in a cohort of women with BD, to (i) investigate sexual function, sexual distress, general sexual satisfaction and QoL; (ii) explore whether sexual distress was related to affective symptoms and (iii) investigate whether QoL was associated to sexual distress.

## Methods

### Study design

Women with BD were recruited to the questionnaire survey from the waiting room at the Copenhagen Affective Disorder Clinic, Psychiatric Centre Copenhagen. The included sample was derived from 1 March to 9 May 2015 from patients attending a region-wide secondary service for patients with primary bipolar disorders. The patients received written and oral information about the project before deciding whether to participate. Further, at the time the questionnaire was answered and diagnosis confirmed, medication was assessed from the patients’ medical records.

The self-assessment questionnaire included parameters, among others, previously shown to affect sexual function: basic demographic information (age, education, civil status, sexual orientation, length of relationship, employment and information about children) and lifestyle (alcohol, smoking and weight). The other part of the questionnaire included is described below.

### Participants

The inclusion criteria were as follows: being women with a clinical diagnosis of BD, aged 18–70 years, reading and understanding Danish and willing to participate in the study. Participants were included in analyses independent of their answers, sexual preference, civil status, age and sexual activity. They were excluded from analysis when not diagnosed with BD according to their electronic hospital records.

The control group consisted of a group of Danish women from a previous study conducted by our group (Giraldi et al. 2015) describing sexual function. In summary, these data were collected from a cross-sectional study of a large, broadly sampled, non-clinical population cohort of Danish women (573 women participated). They were drawn randomly from the Danish Central National Register and invited to participate by letter. The Danish women were included if they were sexually active with a partner within the 4 weeks prior (*n* = 429). As the control group had a significant higher mean age, we conducted an age-matched analysis between women with BD and a subsample of age-matched women from the control group (*n* = 122).

### Sexual function

The Female Sexual Function Index (FSFI) and Female Sexual Distress Scale (FSDS) were used to describe sexual function among the Danish women in the background population. They were categorised as having female sexual dysfunction (FSD) when FSFI score was ≤26.55 (impaired sexual function) and FSDS score ≥15 (Giraldi et al. 2015). In the present cohort, female sexual dysfunction was defined as a CSFQ-14 score ≤41 (impaired sexual function) and FSDS score ≥15 (distress).

### Questionnaires

#### WHOQoL-BREF, Quality of Life measure

Quality of life was assessed using an abbreviated version of the World Health Organisation Quality of Life Assessment brief version, WHOQoL-BREF (WHOQOL Group 1998), Danish version. The WHOQoL-BREF is a 26-item questionnaire developed from the original 100-item questionnaire the WHOQoL-100. The WHOQoL-BREF covers four different subscales (WHOQoL Group 1998): physical health (energy and fatigue, pain and discomfort, sleep and rest), psychological health (bodily image and appearance, negative feelings, positive feelings, self-esteem, thinking, learning, memory and concentration), social relationships (personal relationships, social support and sexual activity) and environment (e.g. financial resources, freedom, physical safety and security, health and social care). Besides, one global item scores overall quality of life (overall QoL) and one global item scores general health. Each individual item of the WHOQoL-BREF is scored from one to five on a response scale. The scores are then transformed linearly to a 0–100 scale except items one and two, overall QoL and general health which are displayed in raw scores 1–5 (Noerholm et al. 2004). Higher scores indicate better QoL. The internal consistency of the Danish WHOQoL-BREF was, in a pilot study, found to be psychometrically valid (Nørholm and Bech 2001). Finally, satisfaction with sexual life was assessed from item 21 (WHOQoL-BREF, “How satisfied are you with your sexual life?”) and categorised as unsatisfied when answered as “very unsatisfied” or “unsatisfied”.

#### Altman Self-Rating Mania Scale (ASRM)

Manic symptom scale scores were assessed by a short 5-item questionnaire that covered the symptoms of positive mood, self-confidence, sleep patterns, speech patterns/amount and motor activity. Each item is scored from 0 to 4 and depends on how the participants described their symptoms over the past week. Scores above five are indicative of mania or hypomania with the severity of symptoms increasing with higher scores (Altman et al. 1997).

#### Major (ICD-10) Depression Inventory (MDI)

Depression was measured by a 10-item questionnaire developed to measure DSM-IV and ICD-10 diagnoses of moderate to severe depression by self-reported symptoms during the past 2 weeks. The first three items cover the core symptoms of depression according to ICD-10 (depressed mood, lack of interest and lack of energy) while the other items cover the accompanying symptoms (lack of self-confidence, self-blame or guilt, thoughts of death or suicide, difficulty thinking and concentrating, agitation or inhibition, sleep disturbances and appetite and weight change). Each item has a possible score between 0 and 5 (0 = at no time, 5 = all the time). Items 8 and 10 were divided into two sub-items and only the highest scores of these items were included in the analysis. The theoretical score was 50 with a range from 0 (no depression) to 50 (major depression) and a higher score indicating the severity of depression. Cut-off scores ≥26 indicate moderate to severe depression. The internal and external validity were found to be psychometrically valid in two Danish studies (Bech et al. 2001; Olsen et al. 2003).

#### Changes in Sexual Functioning Questionnaire-14 (CSFQ-14)

Sexual function was assessed by a questionnaire containing 14 questions with a total cut-off score ≤41 indicating impaired sexual function. Each item is scored in a 5-point scale that refers either to frequency (“never” to “every day”) or to satisfaction (“none” to “great”). The first item scores overall pleasure with present sexual life and the other items cover five subscales (sexual pleasure, sexual desire/frequencies, sexual desire/interest, arousal/excitement and orgasm), and two items (10 + 14) about loss of arousal and painful orgasm (de Boer et al. 2014; Keller et al. 2006). CSFQ-14 has been validated to assess sexual dysfunction among patients taking antipsychotics and also covers all stages of sexual functioning compared to other questionnaires (de Boer et al. 2014). A score ≤41 is seen as an impaired sexual function and thus indicates sexual dysfunction (Keller et al. 2006).

#### Female Sexual Distress Scale (FSDS)

Sexual distress was assessed by a 12-item questionnaire that covers the intensity and frequency of sexual distress the past 30 days. Each item scores from 0 to 4 (0 = never and 4 = always). A cut-off score ≥15 indicates sexual distress. FSDS has been validated in studies, including a pilot study with 60 healthy women and 18 women with different kinds of sexual dysfunction. It was concluded that the questionnaire is able to distinguish between those with and without sexual dysfunction and the study showed moderate positive correlation to general mental distress, and thus seems to be a good estimate of sexual distress (Derogatis et al. 2002).

### Ethical statement

Written informed consent was obtained from all participants. The study was approved by the Danish Data Protection Agency, Journal Number: 03568 and ID-Number: RHP-2015-004.

### Statistical analysis

All analyses were conducted using the Statistical Package for Social Sciences (version 22.0). Spearman’s correlation coefficients were used to test the associations between the variables (data measured from an ordinal scale). Chi square tests were used to compare variables within two groups and prevalence values between two groups. The study population (women patients with BD), Danish women from the background population as well as the age-matched controls were, separately, dichotomised into two groups: sexually distressed or not sexually distressed. An independent *t* test was used to compare mean scores and standard deviations. To investigate whether MDI scores or WHOQoL subscales were predictive of sexual distress, binary logistic regression analyses including age and depression score according to MDI and the five WHOQoL subscales (general health, physical health, psychological health, social relationships and environment), were conducted, respectively.

Finally, we compared information on sexual function among women with BD with data from women from the Danish background population and age-matched controls from this group, respectively.

## Results

### Study characteristics

In total, 85 women were asked to participate; 14 did not answer the questionnaires (response rate 83.5%), eight were not diagnosed with BD and a further two did not fully complete the questionnaires, thus leaving 61 participants (72% included). As seen from Table 1, the participants were characterised by being young, single without children, heterosexual, well-educated and unemployed. The majority were non-smokers and overweight to obese (BMI > 25).

**Table 1:** Demographic data and background characteristics of women with BD, *N* = 61, standard deviation I brackets

Age (range 19–63 years)	33.7 (10.0)
Education	
No training	7 (12%)
Higher education	38 (62%)
Other	16 (26%)
Employed	19 (31%)
Sexual orientation	
Heterosexual	50 (82%)
Homosexual/bisexual	11 (18%)
Civil status	
Single	28 (46%)
Married	7 (11%)
Cohabiting with partner	11 (18%)
In a relationship, but live apart	15 (25%)
Children	23 (38%)
Children living at home	18 (30%)
Current smoker	21 (34%)
Menopause	5 (8%)
Body mass index (BMI = kg/m^2^); mean ± SD	26 (6)
BMI < 18.5 (underweight)	2 (3%)
BMI 18.5–25 (normal weight)	28 (46%)
BMI > 25 (overweight to obesity)	31 (51%)
Medication	
Antidepressants	8^a^ (13%)
Antipsychotics	35 (57%)
Sleeping pills/benzodiazepines	24 (39%)
Mood stabilisers/lithium	50 (82%)
No medication	4 (7%)
Affective symptoms	
ASRM	10 (16%)
MDI	20^b^ (33%)
In remission	31 (51%)
WHOQoL-BREF; mean ± SD	
Overall QoL	3.3 (0.9)
General health	2.0 (1.1)
Physical health	62.7 (17.5)
Psychological health	26.9 (19.8)
Social relationships	58.7 (22.4)
Environment	62.6 (14.6)

Altman Self-Rating Mania Scale (ASRM) number of patients with current hypomania/manias defined as a score >5. Major Depression Inventory (MDI) number of patients with current depression defined as a score ≥26 (moderate/severe). Remission defined as ASRM scores <5 and MDI score <26

*WHOQoL-BREF* World Health Organisation Quality of Life-BREF

^a^Five patients were prescribed selective serotonin reuptake inhibitors (SSRI) and serotonin and noradrenaline reuptake inhibitors (SNRI), one patient a combination of SNRI and Agomelatin and two patients Agomelatin

^b^Five patients with depression also reported ASRM scores with a mean score ASRM = 10

Almost all were prescribed medication, especially mood stabilisers (mean number of medication: 2.2), and half were in remission. Women with BD and BMI > 25 were not prescribed more types of medication (mean 2.1) than those with BMI < 25 (mean 2.3). Four women (6.6%) did take lithium in combination with benzodiazepines and three of them reported sexual dysfunction.

Most women were sexually active (87%). All the sexually inactive (*n* = 8) women reported impaired sexual function but three of them did not report sexual distress. Half of the sexually inactive women with BD were older than 50 years, single or in a relationship for many years (25 or 31 years). Four women with BD were older than 50 years old and they all reported impaired sexual function but we did not find a correlation between a lower CSFQ-14 score and higher age (*r* = −0.17, *P* = 0.20).

### Impaired sexual function, sexual distress and general sexual satisfaction—prevalence, mean scores and correlation to affective symptoms

As seen from Table 2, 21 (34.4%) women with BD reported impaired sexual function (CSFQ-14 score ≤41). However, six patients were not distressed by this resulting in 15 (24.6%) out of 21 patients having a sexual dysfunction (impaired sexual function combined with sexual distress). Thirty-three (54.1%) women with BD had sexual distress. More than one-third was unsatisfied (answering “very unsatisfied” or “unsatisfied” to the question “How satisfied are you with your sexual life?”) with their sexual life. The study population had a mean score ± SD of CSFQ-14 score of 45.2 ± 10.4; FSDS score 15.9 ± 11.3; ASRM scores 3.7 ± 3.8 and MDI score 19.7 ± 12.1.

**Table 2:** Sexual dysfunction and sexual distress

	Study population (*n* = 61)	Background population (*n* = 429)	*P* (study and background)	Age-matched (*n* = 122)	*P* (study and age-matched)
Sexual dysfunction	15 (24.6)	72 (16.8)	0.19	25 (20.5)	0.53
Impaired sexual function (FSFI or CSFQ-14)	21 (34.4)	140 (32.6)	0.89	45 (36.9)	0.74
Sexual distress (FSDS)	33 (54.1)	115 (26.8)	0.001	46 (37.7)	0.04
Unsatisfied with sexual life^a^	24 (39.3)	86 (20.0)	0.001	–	–

Prevalence and comparison between women with bipolar disorder and women from the Danish background population including age-matched control persons

Data are prevalence (%). Data from the background population is from a Danish study (Giraldi et al. 2015). FSFI =  Female Sexual Function Index (background population, impaired sexual function cut-off ≤26.55). CSFQ-14 = Changes of Sexual Functioning Questionnaire (study population, impaired sexual function cut-off score ≤41). FSDS = Female Sexual Distress Scale (score ≥15 = sexual distress). Sexual dysfunction (FSFI cut-off ≤26.55 or CSFQ cut-off score ≤41 combined with FSDS score ≥15)

^a^Answering “very unsatisfied” or “unsatisfied” to “How satisfied are you with your sexual life?” In background population: “bad” or “very bad” to “How do you appraise your sexual life?”

Using Spearman’s correlation, sexual function (CSFQ-14 score) was positively correlated with ASRM scores (*r* = 0.26, *P* = 0.04) but not with MDI scores (*r* = −0.02, *P* = 0.88). However, sexual distress was positively correlated with MDI scores (*r* = 0.33, *P* = 0.01) but not with ASRM scores (*r* = 0.11, *P* = 0.42). Thus overall, general sexual satisfaction did not correlate with affective symptom scale scores (ASRM scores *r* = 0.13, *P* = 0.32; MDI scores *r* = −0.21, *P* = 0.10).

### Comparison with the background population

As seen from Table 2, women with BD did not have a significantly higher prevalence of impaired sexual function or sexual dysfunction compared to Danish women from the background population but they were significantly more often sexually distressed (*P* = 0.001, *n* = 429), also when using an age-matched subsample from the background population (*P* = 0.04, *n* = 122). Age-matched controls (*n* = 122) also had a lower BMI than the study population but almost equal percentage of women in menopause (8% vs. 7%).

### Quality of life

Sexual function was positively associated with QoL and social relationships. Better sexual function and less sexual distress was associated with a higher degree of satisfaction with sexual life (*r* = 0.51, *P* = 0.00, *r* = −0.32, *P* = 0.01) and higher QoL score (Table 3), respectively. Finally, QoL scores, including the four subscales of WHOQoL, were negatively correlated with MDI scores.

**Table 3:** Spearman’s correlations between WHOQoL-BREF scores, affective symptoms and sexual function and distress in a cohort of women with bipolar disorder

	Mania scale (ASRM)	Depression (MDI)	Sexual function (CSFQ-14)	Sexual distress (FSDS)	Sexual satisfaction (item 21)
QoL	0.22 (0.09)	−0.55 (0.00)	0.24 (0.06)	−0.25 (0.06)	0.33 (0.01)
Health	0.21 (0.10)	−0.49 (0.00)	0.20 (0.13)	−0.23 (0.07)	0.23 (0.08)
Physical health	−0.05 (0.70)	−0.70 (0.00)	−0.04 (0.78)	−0.21 (0.11)	0.21 (0.10)
Psychological health	0.03 (0.82)	−0.77 (0.00)	0.16 (0.21)	−0.38 (0.00)	0.36 (0.00)
Social relationships	0.08 (0.54)	−0.36 (0.00)	0.40 (0.00)	−0.17 (0.20)	0.76 (0.00)
Environment	−0.18 (0.16)	−0.39 (0.00)	0.10 (0.43)	−0.13 (0.33)	0.14 (0.29)

*R* and *P* values (in brackets)

*WHOQoL-BREF* World Health Organisation Quality of Life-BREF, *ASRM* Altman Self-Rating Mania Scale, *MDI* Major Depression Inventory, *FSDS* Female Sexual Distress Scale, *CSFQ-14* Changes of Sexual Functioning Questionnaire

In post hoc analyses dividing the women with BD into two groups—not sexually distressed and sexually distressed—the sexually distressed group had significantly lower scores for psychological health and a trend towards higher depression score according to MDI (*P* = 0.07) (Table 4). In further analyses exploring the influence of MDI scores on the level of reported sexual distress, a multiple linear regression analysis adjusted for age and self-reported depressive symptoms according to MDI was conducted and showed that the MDI score was borderline associated with being sexually distressed [*P* = 0.06, Wald = 3.6, exp(B) = −0.04 (CI 0.92–1.00)]. In further regression analyses, the WHOQoL subscales were added and none of these were associated with a significant higher level of sexual distress: general health: *P* = 0.70, Wald = 0.15, exp(B) = 0.99 (CI 0.95–1.03); psychological health: *P* = 0.33, Wald = 0.97, exp(B) = 1.02 (CI 0.98–1.07); social relationships: *P* = 0.95, Wald = 0.003, exp(B) = 1.01 (CI 0.98–1.03); environment: *P* = 0.71, Wald = 0.14, exp(B) = 0.97 (CI 0.97–1.05), respectively.

**Table 4:** Comparison of mood symptoms and quality of life dividing the dividing the cohort of women with bipolar disorder according to the Female Sexual Distress Scale (score <15 = not sexually distressed and score ≥15 = sexual distressed)

	Not sexually distressed (*n* = 28)	Sexually distressed (*n* = 33)	*P*
Mean (SD)			
Age	31.9 (8.3)	33.5 (11.3)	0.54
ASRM	3.4 (4.0)	3.9 (3.6)	0.52
MDI	16.6 (12.2)	22.4 (11.6)	0.07
BMI	26.8 (5.6)	26.2 (7.8)	0.77
WHOQoL-BREF			
Quality of life	3.5 (0.8)	3.1 (0.9)	0.07
General health	3.2 (1.0)	2.8 (1.1)	0.15
Physical health	65.1 (19.3)	60.6 (15.9)	0.33
Psychological health	52.6 (19.8)	42.1 (18.8)	0.04
Social relationships	61.4 (21.8)	56.4 (23.0)	0.39
Environment	64.6 (14.2)	60.9 (15.0)	0.33
Number (%)			
Menopause	2 (7.1)	3 (9.1)	0.78
In a relation^a^	13 (46.4)	20 (60.6)	1.72
Sexually active	25 (89.3)	28 (84.8)	0.90

ASRM scores defined as Altman Self-Rating Mania Scale (ASRM) score >5. Depression defined as Major Depression Inventory (MDI) score ≥26

*SD* standard deviation, *BMI* body mass index, *WHOQoL-BREF* World Health Organisation Quality of Life-BREF, *CSFQ-14* Changes of Sexual Functioning Questionnaire score ≤41)

^a^Married, cohabiting with a partner or in a relationship but living apart

## Discussion

The present study investigated sexual dysfunction, sexual distress, general sexual satisfaction and QoL in a cohort of women with BD and whether sexual distress was related to affective symptoms and QoL. Overall, 61 women with BD were included; one-fourth had a sexual dysfunction (an impaired sexual function combined with sexual distress), more than half of the study population was sexually distressed and one-third was unsatisfied with their sexual life. Better sexual function was related to higher ASRM scores while sexual distress and lower OoL score were associated to higher MDI scores.

In comparison, an Italian study (142 patients, 60 with BD and 82 with unipolar depression) found a higher prevalence of sexual dysfunction among men and women with BD compared to patients with unipolar depression and healthy control persons (*n* = 101) (Dell’Osso et al. 2009). Another population-based Danish study (*n* = 4415, 2295 women) found that 11% of Danish women had at least one sexually related problem (lubrication, anorgasmia, dyspareunia and/or vaginismus) considered as a problem (Christensen et al. 2011a). The latter study did not include problems with sexual desire which might have underestimated the prevalence of sexual dysfunction since it is the most common sexually related problem (Hayes et al. 2006). In comparison with the Danish background population included as comparison group in the present study, the women with BD did not have a significantly higher prevalence of sexual dysfunction but they reported sexual distress more often. Anyhow, it can be difficult to compare the prevalence of sexual dysfunction across studies due to different definitions of sexual dysfunction and varying time frames in questionnaires (Hayes et al. 2006; Giraldi et al. 2011).

One might have expected a higher prevalence of sexual dysfunction among women with BD; however, some of the women in the background population might have been diagnosed with BD as well. We did include age as a potential confounder in the regression analyses. When compared with the entire group of women in the Danish background population (20–65 years) (Giraldi et al. 2015), the present study cohort (19–63 years) was younger and the prevalence of sexual dysfunction was more equal among age-matched control persons and women with BD than compared to all women in the Danish background population. It seemed that higher age did not predict sexual dysfunction in the background population even though the risk of developing sexual dysfunction is higher below 30 years or above 50 years of age (Christensen et al. 2011a) and additionally, lower sexual desire is also associated with increasing age (Eplov et al. 2007).

Sexual distress was negatively associated with sexual satisfaction and distress was associated with depressive symptom scale scores in line with other studies (Hayes et al. 2008). More than half of the present study population was unsatisfied with their sexual life and it was not related to MDI or ASRM scores. Sanders et al. (2011) found that worries about sexual life increased the risk of developing an impaired sexual function. This is in line with a study reporting that less distress was reported among those who were better at communicating their sexual needs (Hayes 2008).

Quality of life (overall, general health and the four subscales) was associated with higher MDI scores. Better sexual function was associated with higher scores for QoL overall, and for social relationships, including item 21 also (“How satisfied are you with your sexual life?”), separately. Not surprisingly, those with better sexual function also seemed more satisfied with their sexual life. In comparison with the Danish background population (a study including 568 women), the present cohort had lower scores on the four WHOQoL subscales (physical health: 75 ± 19 vs. 62.7 ± 17.5, psychological health: 67 ± 17 vs. 46.9 ± 19.8, social relationships: 70 ± 18 vs. 58.7 ± 22.4 and environment: 74 ± 16 vs. 62.6 ± 14.6) (Noerholm et al. 2004). This is in line with other studies where patients with affective disorders were found to have lower quality of life scores (Nørholm 2008; Yatham et al. 2004). Sexual distress was related to a lower overall QoL score and for psychological health. Dichotomising the study population into sexually distressed and not sexually distressed groups, no difference was found between their scores for social relationships but psychological health differed. This may be explained by the influence of psychopathology related to BD rather than social relationships that may have had influence on QoL in a negative way. The two groups did not differ according to age, BMI and MDI scores (although MDI scores showed a trend, Table 4). Those who were sexually distressed reported higher MDI scores which included negative thoughts about oneself, reduced self-esteem, problems with memory and concentration. It may partly contribute to the lower score on psychological health reported by the sexually distressed women with BD.

The study population had a higher BMI compared to the age-matched controls. The majority of women with BD were prescribed antipsychotics and/or mood stabilisers, frequently quetiapine, lamotrigine and/or lithium (Table 1). Weight gain is a well-known side effect of antipsychotics and lithium which might have contributed to more than half of the study population being overweight or obese (BMI > 25, Table 1). High BMI is associated with impaired sexual function and sexual inactivity (Christensen et al. 2011c). It is difficult to distinguish whether women who are obese (BMI ≥ 30) and diagnosed with BD had sexual dysfunction due to their psychiatric diagnosis, side effects of medication and/or because of their obesity or other factors. However, the obese participants were not prescribed more medicine than the rest of the cohort.

Quetiapine seems to be associated with less sexually related side effects than other antipsychotics and lamotrigine seems not to be related to sexual dysfunction (Serretti and Chiesa 2011a, b). In a study with only 15 women with BD, it was estimated that around 50% who started treatment with lithium developed a sexual dysfunction (Grover et al. 2014), while another study (including *n* = 105, 59 women with BD) concluded that lithium monotherapy did not present a risk factor for sexual dysfunction only when combined with benzodiazepines (Ghadirian et al. 1992). In the present study, four women did take lithium in combination with benzodiazepines and three of them had sexual dysfunction. Since they were taking more types of medication it may be assumed that these patients were difficult to treat and thereby also more clinically unstable with an impact on sexual function. Finally, it is well known that selective serotonin reuptake inhibitors (SSRI) and serotonin and noradrenaline reuptake inhibitors (SNRI) often have side effects, especially anorgasmia (Bergh and Giraldi 2014). However, only one of the sexually inactive women having problems with orgasm in the present study was prescribed an SSRI or SNRI but the remaining five patients (sexually active) taking SSRI and/or SNRI medication also reported problems with orgasm.

Menopause may be a risk factor for sexual dysfunction. All the post-menopausal women with BD reported impaired sexual function. In line with that, a Spanish study (*n* = 260) found that menopause was associated with a lower score for CSFQ-14 (worse sexual function) (Ornat et al. 2013) but in this study no association was found between a lower score of CSFQ-14 and higher age.

In the present study, more women with BD identified themselves as homo- or bisexual (18%, Table 1) in comparison with the Danish background population (1.6%) (Graugaard et al. 2015). According to the latter study, non-heterosexual Danes reported more often that their sexual needs were not met, which might contribute to sexual distress. It is of interest that more women with BD identify themselves as non-heterosexual in comparison to the general population and that they have sexual distress to a higher extent. However, these questions are beyond the limits of the present study and need larger study samples to answer them.

## Strengths and limitations

This study was limited by the cross-sectional design and a questionnaire survey does not fully explain the causations of sexual function, affective symptoms and quality of life. The study included women only and it is therefore recommended in future studies to include both sexes. However, it is the first study including sexual distress in a definition of sexual dysfunction among women with BD. There was a high response rate (84.7%) which might have minimised the risk of selection bias towards women with BD without manic or depressive symptoms—they might be more likely to answer the questionnaire. The study included clinical data and the questionnaires were validated in order to estimate sexual function, sexual distress, affective symptoms and quality of life. Unfortunately, time frames varied between the questionnaires, and as it was not possible to change this in the present validated form, it represents a limitation.

It was a strength that age was included as a potential confounder due to the data being age- matched when comparing the prevalence of sexual dysfunction and distress among women with BD and Danish women from the background population. The present study was limited by not being matched according to sexual activity and civil status as women with BD were not excluded if sexually inactive (13%) or not in a current relationship (46%). If we had excluded the sexually inactive women, the prevalence of impaired sexual function would have been lower. It is further a limitation that the cross-sectional design of the present study did not allow us to explore whether they were inactive because of a present sexual dysfunction or whether they had a sexual dysfunction due to sexual inactivity at the time of answering the questionnaire—they might not have had problems with orgasm, desire or lubrication. Finally, as described, the psychopharmacological treatment can induce sexual dysfunction and only 7% of the included women were without any medication. Due to the low number of participants it was not possible to correlate sexual function and the different kinds of medication.

## Conclusions

Women with BD reported a high prevalence of sexual distress (more than 50%) and significantly higher than age-matched Danish women; more than one-third were unsatisfied with their sexual lives. Further, sexual function seemed related to higher manic scores (positive correlation) while sexual distress was negatively correlated with higher depressive scores. QoL scores were negatively correlated with depression scores. Further, women reporting sexual distress had lower scores on the QoL subscale for psychological health, thus suggesting that the impact of BD influences overall QoL. Finally, the present findings point to the fact that sexual distress can be an expression of depression.

However, in line with a recent review (Kopeykina et al. 2016), although patients with BD experience disease-specific impaired sexual functions, the existing literature on patients with BD sexual behaviour is mostly outdated. Novel research is thus needed to address sexual symptomatology in BD and longitudinal studies are important to disentangle the causal relationship between sexual distress, affective symptoms and QoL.

## Abbreviations

ASRM: Altman Self-Rating Mania Scale
BD: bipolar disorder
BMI: body mass index
DSM-V: Diagnostic and Statistical Manual of Mental Disorders, fifth version
CSFQ-14: Changes in Sexual Functioning Questionnaire
FSDS: Female Sexual Distress Scale
MDI: Major (ICD-10) Depression Inventory
QoL: quality of life
WHOQoL-BREF: World Health Organisation Quality of Life Assessment, brief version

## References

[CR1] Altman EG, Hedeker D, Peterson JL, Davis JM (1997). The Altman Self-Rating Mania Scale. Biol Psychiatry.

[CR2] Basson R, Berman J, Burnett A, Derogatis L, Ferguson D, Fourcroy J (2000). Report of the international consensus development conference on female sexual dysfunction: definitions and classifications. J Urol.

[CR3] Basson R, Leiblum S, Brotto L, Derogatis L, Fourcroy J, Fugl-Meyer K (2004). Revised definitions of women’s sexual dysfunction. J Sex Med.

[CR4] Basson R, Wierman ME, van Lankveld J, Brotto L (2010). Summary of the recommendations on sexual dysfunctions in women. J Sex Med.

[CR5] Bech P, Rasmussen NA, Olsen LR, Noerholm V, Abildgaard W (2001). The sensitivity and specificity of the major depression inventory, using the Present State Examination as the index of diagnostic validity. J Affect Disord.

[CR6] Bergh SJ, Giraldi A (2014). Sexual dysfunction associated with antidepressant agents. Ugeskr Laeger.

[CR7] Brotto LA, Petkau AJ, Labrie F, Basson R (2011). Predictors of sexual desire disorders in women. J Sex Med.

[CR8] Christensen BS, Grønbaek M, Osler M, Pedersen BV, Graugaard C, Frisch M (2011). Sexual dysfunctions and difficulties in Denmark: prevalence and associated sociodemographic factors. Arch Sex Behav.

[CR9] Christensen BS, Grønbaek M, Osler M, Pedersen BV, Graugaard C, Frisch M (2011). Associations between physical and mental health problems and sexual dysfunctions in sexually active Danes. J Sex Med.

[CR10] Christensen BS, Grønbaek M, Pedersen BV, Graugaard C, Frisch M (2011). Associations of unhealthy lifestyle factors with sexual inactivity and sexual dysfunctions in Denmark. J Sex Med.

[CR11] Clayton AH, El Haddad S, Iluonakhamhe J-P, Ponce Martinez C, Schuck AE (2014). Sexual dysfunction associated with major depressive disorder and antidepressant treatment. Expert Opin Drug Saf.

[CR12] de Boer MK, Castelein S, Wiersma D, Schoevers RA, Knegtering H (2014). A systematic review of instruments to measure sexual functioning in patients using antipsychotics. J Sex Res.

[CR13] Dell’Osso L, Carmassi C, Carlini M, Rucci P, Torri P, Cesari D (2009). Sexual dysfunctions and suicidality in patients with bipolar disorder and unipolar depression. J Sex Med.

[CR14] Derogatis LR, Rosen R, Leiblum S, Burnett A, Heiman J (2002). The Female Sexual Distress Scale (FSDS): initial validation of a standardised scale for assessment of sexually related personal distress in women. J Sex Marital Ther.

[CR15] Eplov L, Giraldi A, Davidsen M, Garde K, Kamper-Jørgensen F (2007). Sexual desire in a nationally representative Danish population. J Sex Med.

[CR16] Ghadirian AM, Annable L, Bélanger MC (1992). Lithium, benzodiazepines, and sexual function in bipolar patients. Am J Psychiatry.

[CR17] Giraldi A, Kristensen E, Sand M (2015). Endorsement of models describing sexual response of men and women with a sexual partner: an online survey in a population sample of Danish adults ages 20–65 years. J Sex Med.

[CR18] Giraldi A, Rellini A, Pfaus JG, Bitzer J, Laan E, Jannini EA (2011). Questionnaires for assessment of female sexual dysfunction: a review and proposal for a standardised screener. J Sex Med.

[CR19] Graugaard C, Giraldi A, Frisch M, Falgaard Eplov L, Davidsen M (2015). Self-reported sexual and psychosocial health among non-heterosexual Danes. Scand J Public Health.

[CR20] Grover S, Ghosh A, Sarkar S, Chakrabarti S, Avasthi A (2014). Sexual dysfunction in clinically stable patients with bipolar disorder receiving lithium. J Clin Psychopharmacol.

[CR21] Hayes RD (2008). Assessing female sexual dysfunction in epidemiological studies: why is it necessary to measure both low sexual function and sexually-related distress?. Sex Health.

[CR22] Hayes RD, Bennett CM, Fairley CK, Dennerstein L (2006). What can prevalence studies tell us about female sexual difficulty and dysfunction?. J Sex Med.

[CR23] Hayes RD, Dennerstein L, Bennett CM, Sidat M, Gurrin LC, Fairley CK (2008). Risk factors for female sexual dysfunction in the general population: exploring factors associated with low sexual function and sexual distress. J Sex Med.

[CR24] Keller A, McGarvey EL, Clayton AH (2006). Reliability and construct validity of the Changes in Sexual Functioning Questionnaire short-form (CSFQ-14). J Sex Marital Ther.

[CR25] Kopeykina I, Kim HJ, Khatun T, Boland J, Haeri S, Cohen LJ, Galynker II (2016). Hypersexuality and couple relationships in bipolar disorder: a review. J Affect Disord.

[CR27] McCabe MP, Sharplip ID, Atella E, Balon R, Fisher AD, Laumann EL (2016). Definitions of sexual dysfunctions in women and men: a consensus statement from the fourth international consultation on sexual medicine 2015. J Sex Med.

[CR26] Noerholm V, Groenvold M, Watt T, Bjorner JB, Rasmussen NA, Bech P (2004). Quality of life in the Danish general population—normative data and validity of WHOQOL-BREF using Rasch and item response theory models. Qual Life Res Int J Qual Life Asp Treat Care Rehabil.

[CR28] Nørholm V (2008). Measuring the quality of life of mentally-ill patients: is it possible to compare with quality of life of healthy population?. Ugeskr Laeger.

[CR29] Nørholm V, Bech P (2001). The WHO Quality of Life (WHOQOL) Questionnaire: Danish validation study. Nord J Psychiatry.

[CR30] Olsen LR, Jensen DV, Noerholm V, Martiny K, Bech P (2003). The internal and external validity of the major depression inventory in measuring severity of depressive states. Psychol Med.

[CR31] Ornat L, Martínez-Dearth R, Muñoz A, Franco P, Alonso B, Tajada M (2013). Sexual function, satisfaction with life and menopausal symptoms in middle-aged women. Maturitas.

[CR32] Sanders SA, Graham CA, Milhausen RR (2011). Predicting impaired sexual functions in women: the relevance of sexual excitation and sexual inhibition. Arch Sex Behav.

[CR33] Serretti A, Chiesa A (2011). Sexual side effects of pharmacological treatment of psychiatric diseases. Clin Pharmacol Ther.

[CR34] Serretti A, Chiesa A (2011). A meta-analysis of sexual dysfunction in psychiatric patients taking antipsychotics. Int Clin Psychopharmacol.

[CR35] Vanwesenbeeck I, Have MT, de Graaf R (2014). Associations between common mental disorders and sexual dissatisfaction in the general population. Br J Psychiatry.

[CR36] Yatham LN, Lecrubier Y, Fieve RR, Davis KH, Harris SD, Krishnan AA (2004). Quality of life in patients with bipolar I depression: data from 920 patients. Bipolar Disord.

[CR37] Zemishlany Z, Weizman A (2008). The impact of mental illness on sexual dysfunction. Adv Psychosom Med.

[CR38] WHOQOL Group (1998). Development of the World Health Organisation WHOQOL-BREF quality of life assessment. Psychol Med.

